# Bilateral endogenous endophthalmitis caused by vancomycin-resistant *Staphylococcus epidermidis* in a neonate

**DOI:** 10.1186/s12348-015-0039-y

**Published:** 2015-04-10

**Authors:** Nidhi Relhan, Thomas Albini, Avinash Pathengay, Harry W Flynn

**Affiliations:** Department of Ophthalmology, Bascom Palmer Eye Institute, University of Miami, Miller School of Medicine, 900 NW 17th Street, Miami, FL 33136 USA; Kode Venkatadri Chowdry Campus, Tadigadapa, Vijayawada, 521137 Andhra Pradesh India; Retina and Uveitis Department, L V Prasad Eye Institute, GMR Varalakshmi Campus, Visakhapatnam, 530040 India

**Keywords:** Endogenous endophthalmitis, Neonatal endophthalmitis, Vancomycin resistance

## Abstract

**Background:**

Neonatal bilateral endogenous endophthalmitis is rare and often results in devastating visual outcome.

**Findings:**

An 18-day-old neonate presented with whitening of the cornea in the left eye. The child was examined under anesthesia, and a diagnosis of bilateral endogenous endophthalmitis was made. Vitreous biopsy from the left eye showed no growth. Blood samples showed growth of *Staphylococcus epidermidis* which was multidrug resistant (including vancomycin) but sensitive to piperacillin-tazobactam. The patient was managed with bilateral intravitreal injections of piperacillin-tazobactam and systemic cefpodoxime. Systemic and topical antibiotics were given for 3 and 8 weeks, respectively, and infection was controlled. At 2-year follow-up, the right eye is fixing and following to light with clear view of the fundus and the left eye has a clear cornea with red glow of the fundus.

**Conclusions:**

Vancomycin-resistant *S. epidermidis* may be a cause of endogenous endophthalmitis. Intravitreal piperacillin-tazobactam and systemic cefpodoxime were used to eliminate the infection in this neonate.

## Findings

### Background

Endogenous endophthalmitis in the pediatric population is rare and is usually associated with poor visual outcomes. Endogenous endophthalmitis in neonates constitutes 0.1% to 4% of all endogenous endophthalmitis cases [1-3], with the highest incidence of cases reported in India and the lowest in the United States. Gram-positive organisms are causative in 30% to 35% of endogenous endophthalmitis [4,5]. Potential risk factors [6,7] for neonatal endogenous endophthalmitis are immune-compromised status, low birth weight (<1,500 g), bacteremia, prematurity, blood transfusion, respiratory infection, perinatal infection, hospital admission, intravenous catheters, and retinopathy of prematurity. Vancomycin resistance among enterococci, coagulase-negative *Staphylococci*, and *Bacillus* is being increasingly reported [8] in ocular infections. Herein, we report bilateral endogenous endophthalmitis due to multidrug-resistant gram-positive cocci in a neonate of age 18 days.

### Case report

**Figure 1:**
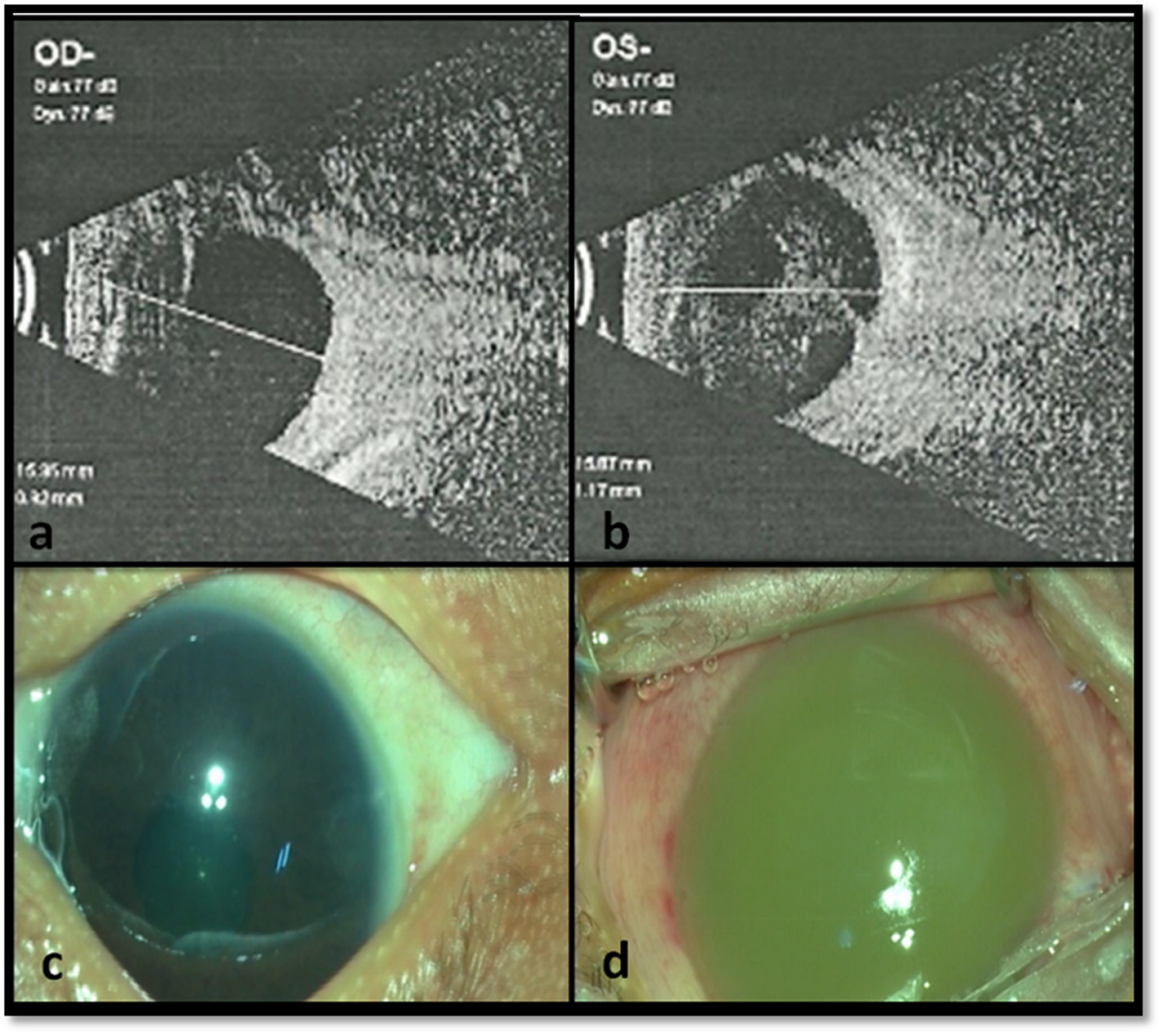
**Ultrasound B-scan and clinical photographs of both eyes taken under operating microscope at presentation.** Ultrasound B-scan shows attached retina in both eyes with clear vitreous cavity in the right eye **(a)** and echogenic shadows in the left vitreous cavity **(b)**. Clinical photographs taken under operating microscope shows clear cornea in the right eye **(c)** and diffuse circumciliary congestion with opaque cornea obscuring any further details in the left eye **(d)**.

An 18-day full-term male baby, born by normal vaginal delivery in rural India, presented with redness in the left eye for 1 day. There was no history of maternal fever, vaginal purulent discharge, or any complication before, during, or after birth. On initial examination, the child looked active, the right eye lacked a red reflex, and the left eye exhibited leukocoria. Echography showed an attached retina in both eyes without any evidence of intraocular calcification or mass lesion (Figure 1a,b). The left eye exhibited moderate-intensity opacities diffusely in the vitreous cavity. Computed tomography scan of the orbits could not be obtained due to financial limitation. On the day of presentation, after discussing the possibility of intraocular malignancy with the parents and obtaining written informed consent, the child was examined under anesthesia. The right eye had a clear cornea, well-formed anterior chamber, non-dilating pupil, iris vascular congestion, clear lens, and vitritis with no fundus view (Figure 1c). In the left eye, conjunctival congestion (deep and diffuse) was seen with diffuse corneal edema obscuring all further details (Figure 1d). A diagnosis of endogenous endophthalmitis was made. In the left eye, the anterior chamber was irrigated with saline through a single limbal paracentesis but it did not alter the corneal appearance which remained opaque white. A vitreous biopsy could only be performed in the left eye with a 23 G vitreous cutter at 0.5 mm from the limbus because of no view to the posterior segment. The sclerotomy port was sutured with 7-0 vicryl suture. Intravitreal antibiotics (vancomycin 0.5 mg/.05 ml + ceftazidime 1 mg/0.05 ml) and dexamethasone (200 μg/0.05 ml) were injected in both eyes with separate 29 G needles at 0.5 mm from the limbus since there were no fungal filaments on smear evaluation of the vitreous. Details of treatment are mentioned in Table 1.

**Table 1:** **Treatment details of the patient**

**Day of presentation (age in days)**	**Events**
1 (18th day of life)	Surgical treatment
	Left eye - anterior chamber wash + vitreous biopsy
	Both eyes - IOAB (V + C + D)^a^
	Topical treatment in both eyes - ciprofloxacin 0.3% four times a day, prednisolone acetate 1% every 3 h, and atropine 1% eye ointment at nighttime^b^
	Systemic treatment - intravenous cefpodoxime by a pediatrician^c^
3 (21st day of life)	Both eyes - IOAB (V + C + D)^a^
8 (26th day of life)	Both eyes - IOAB (P/T + D)^d^
13 (31st day of life)	Left eye - IOAB (P/T + D)^d^

^a^IOAB (V + C + D) - intravitreal antibiotics (vancomycin 0.5 mg/.05 ml + ceftazidime 1 mg/0.05 ml) and dexamethasone (200 μg/0.05 ml); ^b^topical antibiotics were continued for 8 weeks (until resolution of all active inflammation); ^c^systemic antibiotics were given for 3 weeks; ^d^IOAB (P/T + D) - intravitreal antibiotics [piperacillin- tazobactam (112.5 μg/0.05 ml)] and dexamethasone (200 μg/0.05 ml).

Vitreous biopsy was negative for any growth while the blood culture showed gram-positive organisms (*Staphylococcus epidermidis*) which were resistant to vancomycin, amoxicillin, kanamycin, cephalothin, and erythromycin by the Kirby-Bauer disk diffusion method. Urine cultures of the neonate and the mother and culture of a vaginal swab of the mother showed no growth. The isolates were sensitive to piperacillin-tazobactam (112.5 μg/0.05 ml) which was administered intravitreally as mentioned in Table 1. In the subsequent week, the child’s right pupil was mid-dilated with a good view of the disk and macula while the left cornea continued to be opaque with decreased signs of ocular inflammation.

**Figure 2:**
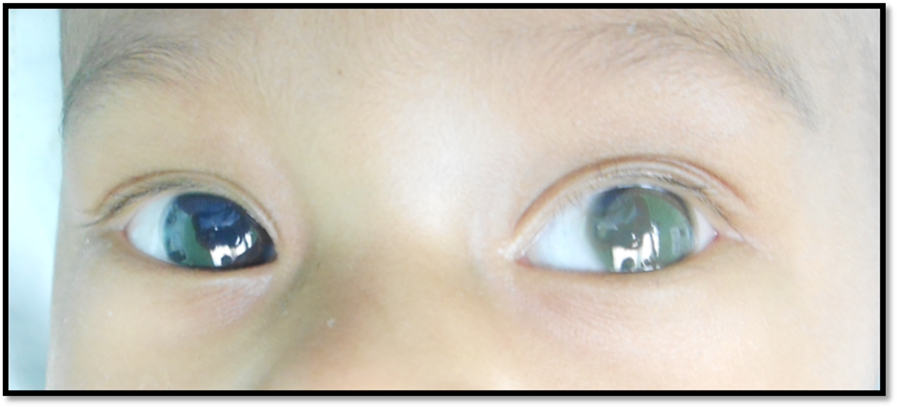
**Clinical picture showing resolved endophthalmitis with clear corneal reflex in both eyes at 1 year follow-up.**

At 12 months of follow-up (Figure 2), no active inflammation was seen in either eye. The right eye fixed and followed light. The corneal whitening in the left eye has resolved. Twenty one months after presentation, cataract surgery was performed on the left eye due to subsequent cataract formation. At the 2-year follow-up, the right eye has good visual function with fix-and-follow vision and a good view of the posterior segment. The left eye is pseudophakic with a good red reflex and has a clear cornea.

### Discussion

Vancomycin resistance among gram-positive bacterial endophthalmitis has become a major global health issue [9]. Vancomycin - a glycopeptide - is the drug of choice for methicillin-resistant coagulase-negative *Staphylococcus* and methicillin-resistant *Staphylococcus aureus* (MRSA) infections [10,11]. Biofilm formation [12] and alteration of binding sites of drug [13] are the mechanisms of acquiring resistance to vancomycin. These resistant traits are then selected for in the presence of vancomycin. The Endophthalmitis Vitrectomy Study in 1996 reported the vancomycin sensitivity of gram-positive organisms to be 100%. A few reports of vancomycin resistance among *Bacillus*, *Enterococcus*, and *Staphylococcus* species causing endophthalmitis have been published recently [11,13,14]. In this patient, the organism was sensitive to piperacillin-tazobactam, cefotaxime, doxycycline, clarithromycin, cefixime, and gentamycin. Piperacillin-tazobactam is a combination of beta-lactam antibiotic with beta-lactamase inhibitor in a ratio of 8:1 (piperacillin/tazobactam) and has broad antibacterial activity against gram-positive and gram-negative pathogens [9,15]. Endogenous endophthalmitis is associated with poor anatomical and visual outcomes. In a study of 26 neonates [6] with endogenous endophthalmitis, 22 out of 26 cases (84.61%) became phthisical. Khera et al. [13] reported a single case of endogenous endophthalmitis, caused by vancomycin-resistant gram-positive cocci, in an adolescent resulting in enucleation. In our case, the child had a better outcome with complete resolution of endophthalmitis in both eyes and achieved fixing and following in the right eye. Maternal infections, sepsis during childbirth, low birth weight, etc. are the reported predisposing factors for neonatal endogenous endophthalmitis. In the present case, detailed history (both mother and child) did not reveal the predisposing factor. The fact that the child was born by a vaginal delivery in the rural India may raise the possibility of infection in perinatal or postnatal period.

In this case, no immunological workup to rule out impaired immunity was done. However, the child responded to the antibiotics well without any further recurrence or disease. Another limitation was that antibiotic resistance was assayed by disk diffusion only and not by minimum inhibitory concentration (MIC) because this assay was not available. Additionally, cultures were positive only from blood and not intraocular fluid; however, the subsequent response to treatment supports our clinical impression that the child’s bacteremia was the cause of endophthalmitis, implicating multidrug-resistant *S. epidermidis* as the target pathogen. To the best of our knowledge, this is the first reported case of vancomycin-resistant bilateral endogenous endophthalmitis in a neonate with good outcomes. This report emphasizes the need of early management including intravitreal antibiotics guided by culture sensitivities which can result in a favorable outcome.

### Conclusion

In conclusion, early recognition of antibiotic resistance and treatment with appropriate antibiotics (as in our case with piperacillin-tazobactam) were associated with resolution of infection. Ocular and extraocular culture and sensitivities should be obtained as early as possible to isolate the causative organism and best chose an appropriate therapy. Appropriate early intervention can result in favorable outcome.

## Consent

The written informed consent from the parents of child was taken before initiating the treatment. IRB of our institute does not require approval to write case report up to 3 cases. 

## Abbreviations

IOAB (V + C + D): Intravitreal antibiotics (vancomycin + ceftazidime + dexamethasone)
IOAB (P/T + D): Intravitreal antibiotics (piperacillin-tazobactam + dexamethasone)

## References

[1] Okada AA, Johnson RP, Liles WC, D’Amico DJ, Baker AS (1994). Endogenous bacterial endophthalmitis. Report of a ten-year retrospective study. Ophthalmology.

[2] Garg SP, Talwar D, Verma LK (1991). Metastatic endophthalmitis: a reappraisal. Ann Ophthalmol.

[3] Aziz HA, Berrocal AM, Sisk RA, Hartley K, Diaz-Barbosa M, Johnson RA, Hess D, Dubovy SR, Murray TG, Flynn HW (2012). Intraocular infections in the neonatal intensive care unit. Clin Ophthalmol.

[4] Jackson TL, Eykyn SJ, Graham EM, Stanford MR (2003). Endogenous bacterial endophthalmitis: a 17-year prospective series and review of 267 reported cases. Surv Ophthalmol.

[5] Wong JS, Chan TK, Lee HM, Chee SP (2000). Endogenous bacterial endophthalmitis: an east Asian experience and a reappraisal of a severe ocular affliction. Ophthalmology.

[6] Jalali S, Pehere N, Rani PK, Bobbili RB, Nalamada S, Motukupally SR, Sharma S (2014). Treatment outcomes and clinicomicrobiological characteristics of a protocol-based approach for neonatal endogenous endophthalmitis. Eur J Ophthalmol.

[7] Moshfeghi AA, Charalel RA, Hernandez-Boussard T, Morton JM, Moshfeghi DM (2011). Declining incidence of neonatal endophthalmitis in the United States. Am J Ophthalmol.

[8] Gupta M, Durand ML, Sobrin L (2011). Vancomycin resistance in ocular infections. Int Ophthalmol Clin Fall.

[9] Yarlagadda V, Akkapeddi P, Manjunath GB, Haldar J (2014). Membrane active vancomycin analogues: a strategy to combat bacterial resistance. J Med Chem.

[10] Roth DB, Flynn HW (1997). Antibiotic selection in the treatment of endophthalmitis: the significance of drug combinations and synergy. Surv Ophthalmol.

[11] Das MK, Pathengay A, Shah GY, Koday NK (2011). Vancomycin-resistant coagulase negative Staphylococcus endophthalmitis following cataract surgery. J Cataract Refract Surg.

[12] Juárez-Verdayes MA, Reyes-López MA, Cancino-Díaz ME, Muñoz-Salas S, Rodríguez-Martínez S, de la Serna FJ, Hernández-Rodríguez CH, Cancino-Díaz JC (2006). Isolation, vancomycin resistance and biofilm production of Staphylococcus epidermidis from patients with conjunctivitis, corneal ulcers, and endophthalmitis. Rev Latinoam Microbiol.

[13] Khera M, Pathengay A, Jindal A, Jalali S, Mathai A, Pappuru RR, Relhan N, Das T, Sharma S, Flynn HW (2013). Vancomycin-resistant Gram-positive bacterial endophthalmitis: epidemiology, treatment options, and outcomes. J Ophthalmic Inflamm Infect.

[14] Pathengay A, Moreker MR, Puthussery R, Ambatipudi S, Jalali S, Majji AB, Mathai A, Husssain N, Dave V, Sharma S, Das T (2011). Clinical and microbiologic review of culture-proven endophthalmitis caused by multidrug-resistant bacteria in patients seen at a tertiary eye care center in southern India. Retina.

[15] Perry CM, Markham A (1999). Piperacillin/tazobactam: an updated review of its use in the treatment of bacterial infections. Drugs.

